# The Role of Mannitol and Vitamin D in Ovarian Ischemia/Reperfusion Injury in Rats with Acute Abdominal

**DOI:** 10.3390/cimb46080526

**Published:** 2024-08-15

**Authors:** Faruk Karateke, Atilla Karateke, Basak Topdagi, Merve Atilgan, Recep Dokuyucu

**Affiliations:** 1 Department of General Surgery, Adana Private Middle East Hospital, 01140 Adana, Turkey; karatekefaruk@hotmail.com; 2 Department of Gynecology and Obstetrics, Private Reyhanlı MMT Amerikan Hospital, 31500 Hatay, Turkey; drkarateke@gmail.com; 3 Department of Dentistry, School of Medicine, Sultan II. Abdulhamid Han Training and Research Hospital, 34668 Istanbul, Turkey; basaktopdagi@gmail.com; 4 Department of Pediatric Surgery, School of Medicine, Necmettin Erbakan University, 42090 Konya, Turkey; atasmerve66@gmail.com; 5 Department of Physiology, Medical Specialization Training Center (TUSMER), 06420 Ankara, Turkey

**Keywords:** total oxidant status (TOS), total antioxidant status (TAS), oxidative stress index (OSI), proliferating cell nuclear antigen (PCNA)

## Abstract

This study was designed to investigate the effects of vitamin D and mannitol in an experimental rat ovarian torsion model. Thirty-two female Wistar albino rats were randomly classified as group 1: (sham), group 2: (detorsion), group 3: (detorsion + mannitol), group 4: (detorsion + vitamin D) and group 5: (detorsion + mannitol + vitamin D) (for each group n = 8). All groups were subjected to bilateral adnexal torsion for 2 h except for group 1. Bilateral adnexal detorsion was performed in all groups except for group 1. Groups 3 and 5 intraperitoneally received the injection of mannitol at a dose of 0.3 mg/kg 30 min before detorsion. Also, the group’s 4 and 5 orally received vitamin D in a dose of 500 IU/kg/day for two weeks before torsion. Total oxidant status (TOS), total antioxidant status (TAS), oxidative stress index (OSI) and proliferating cell nuclear antigen (PCNA) levels were analyzed. According to the histopathological analyses, ovarian tissue damage and follicle counting were evaluated. TOS, OSI and histopathologic score values of ovarian tissue were significantly lower in group 5 than groups 2, 3 and 4 (*p* < 0.05). The PCNA level was significantly higher in group 5 than in groups 2, 3 and 4 (*p* < 0.05). A strong negative correlation was found between OSI and PCNA in groups 2, 3, 4 and 5 (r = −0.92, *p* = 0.01; r = −0.98, *p* < 0.0001; r = −0.98, *p* < 0.0001 and r = −0.96, *p* = 0.0002, respectively). The numbers of primordial follicles in group 5 (*p* < 0.001) and primary follicles in group 4 (*p* < 0.001) were significantly higher when compared to group 2. Based on the results of this study, it could be suggested that combination treatment of mannitol with vitamin D is more effective in reversing tissue damage induced by ischemia–reperfusion (I/R) injury in the ovarian torsion model than administration of only an agent.

## 1. Introduction

Ovarian torsion is defined as a gynecological emergency that is the result of the partial or complete rotation of the ovary around itself and with the side tissues. Ovarian torsion, accounting for 2.7% of all gynecological emergencies, is one of the most common gynecological emergencies in women of reproductive age [1,2]. Indeed, early diagnosis and treatment of ovarian torsion are very important for the preservation of ovarian functions [3]. Additionally, ischemia–reperfusion (I/R) injury is the main pathophysiological problem in ovarian torsion–detorsion [4,5]. Owing to the I/R injury, the ovarian tissues release cytokines, free radicals, neutrophils and nitric oxide and have thrombocyte and apoptosis activation [6,7]. During the I/R period, reactive oxygen species (ROS) are produced, such as hydroxyl radicals, superoxides and hydrogen peroxides [8]. However, the separate determination of all these markers is useless and insufficient in terms of comprehensive evaluation. TAS is used to evaluate the overall antioxidant levels [9]. In addition, TOS is used to determine the all-oxidant levels [1]. OSI is evaluated by the ratio of TOS to TAS and is considered a more accurate indicator of oxidative stress in tissue.

Vitamin D has a significant effect on bone growth and remodeling, and the much broader spectrum activity of vitamin D has been shown in recent studies [10]. Vitamin D and its active form 1–25 (OH)_2_D_3_ (1,25 dihydroxy vitamin D_3_) are involved in important cell functions such as proliferation, differentiation and apoptosis [11,12]. It also has strong antioxidant and anti-inflammatory properties [13]. Previous experimental studies showed the effectiveness of the pretreatment of vitamin D in muscle, kidney and hepatic ischemia and reperfusion [14,15,16,17,18]. Although a lot of agents are used to investigate the treatment of ovarian torsion–detorsion induced by ovary I/R injury in distinctive experimental and clinical models, there has been no previously published experimental study investigating the effect of vitamin D on the ovarian torsion–detorsion induced by ovary I/R injury.

Mannitol has an important role in the pathophysiology of I/R injury through its own free radical scavenging property. This osmotic diuretic is known for its ability to reduce cellular edema and improve microcirculation, which is essential for minimizing tissue damage during the reperfusion phase. Mannitol’s mechanism of action involves the scavenging of reactive oxygen species (ROS), thereby reducing oxidative stress and protecting cellular components from oxidative damage. This makes it a valuable agent in the management of I/R injuries, including those affecting the ovaries [19,20].

PCNA is accepted as a sensitive marker showing the early stages of follicular growth, and also it has also been known that it plays an important role in the development of ovarian follicles. The increase in expression of PCNA in correlation with the proliferation of granulosa cells begins to decrease in the later stages of follicular growth with atresia [21].

Current pharmaceutical treatments for ovarian I/R injury include the use of antioxidants, anti-inflammatory agents (lycopene, curcumin, erdosteine, lipoic acid, vitamin C, melatonin, atorvastatin, methylprednisolone, verapamil, trapidil, etc.) and various other pharmacological interventions aimed at mitigating the effects of oxidative stress and inflammation [1,5,22,23,24,25,26,27]. Despite these advancements, there remains a need for more effective treatments due to the limitations and side effects of existing therapies. New treatments that can offer better protection against oxidative stress and inflammation while minimizing adverse effects are highly desirable.

Although several drugs have been used for treatment of rat ovarian I/R injury to date, no research has evaluated the effectiveness of both mannitol and vitamin D alone and in combination in rat ovarian I/R injury. Because of this, our study is the first investigation into the effects of both mannitol and vitamin D on rat ovarian I/R injury. In the present study, we aimed to investigate the potential protective effects of mannitol and vitamin D in a rat ovary I/R injury model.

## 2. Materials and Methods

### 2.1. Study Design

Thirty-two female adult Wistar albino rats, weighing 260–290 g, were obtained from the Experimental Animal Laboratory of Hatay Mustafa Kemal University. The rats were housed in a climate-controlled room (22 ± 2 °C temperature and 50 ± 5% humidity) on a 12/12 h light/dark cycle with ad libitum food and fresh water. Rats were allowed to adapt to the environment for 5 days before commencing the experiment. The experimental protocol was approved by the animal ethics committee of Mustafa Kemal University (approval number: 2015-1/1). The rats were coincidentally separated equally into 5 groups, which included eight rats in each group. Animals were classified as group 1: (sham), group 2: (detorsion), group 3: (detorsion + mannitol), group 4: (detorsion + vitamin D) and group 5: (detorsion + mannitol + vitamin D).

### 2.2. Experimental Procedures

All rats were weighed and anesthetized by administering a combination of 12 mg/kg xylazine (Rompun, Bayer, Leverkusen, Germany) and 80 mg/kg ketamine (Ketalar, Pfizer, Ann Arbor, MI, USA) intramuscularly. After pre-operative sterilization, a 2 cm midline incision was made in the lower abdomen. In group 1 (Sham operation), the uterine horns and adnexa were observed for 1 min before closing the incision with a 3-0 polyglycolic acid suture. Two hours later, a relaparotomy was performed, and both ovaries of the rats were excised. For the adnexal torsion (AT) technique, the adnexa, including ovarian vessels and tubes, were rotated 360° clockwise and then fixed to the abdominal wall [1,28]. Groups 2 and 3 were subjected to both the AT (2 h of ischemia) and adnexal detorsion (AD) (2 h of reperfusion) techniques. After a total 4 h period, both ovaries were removed through relaparotomy. Only group 3 received mannitol (0.3 mg/kg of a 20% solution) via the inferior caval vein before 30 min AD [24]. In group 4, vitamin D was given orally at a dose of 500 IU/kg daily for two weeks before AT [29]. After the procedure, both adnexal torsion (2 h of ischemia) and adnexal detorsion (2 h of reperfusion) were performed, and both ovaries were excised. Group 5 received 500 mg/kg of vitamin D orally daily for two weeks before AT. Following vitamin D administration, both AT and AD were performed, and mannitol (0.3 mg/kg of a 20% solution) was administered via the inferior caval vein 30 min before AD. After these procedures, both ovaries were removed. Blood samples were collected by cardiopuncture, and the ovaries were placed in a 10% formaldehyde solution for histological examination.

Mannitol (Biofleks 20% mannitol injectable solution) and vitamin D (DEVIT-3 ampul) were obtained from Polipharma and Deva, Istanbul, Turkey, respectively.

### 2.3. Measurements

The ovarian tissues were fixed in a 10% neutral buffered formalin solution for 48 h, and each ovary tissue was embedded in paraffin blocks. Then, the paraffin blocks were sectioned at a thickness of 4 µm by using a microtome (Leica RM2125RTS). Each ovary section was soaked in xylene for deparaffinization. Following the deparaffinization, tissues were subjected to rehydration. After these steps, all sections were stained with hematoxylin-eosin for examination with light microscopy (Olympus Clinical Microscope BX45). At least five microscopic areas were evaluated to assess the scores of the specimens semi-quantitatively. Histopathological changes were graded with regard to particular parameters, including inflammatory cell infiltration, vascular congestion, hemorrhage and cellular degeneration, for all sections. The severity of ovary injury was assessed using an ordinal grading system that ranges from 0 to 3 (0: none; 1: mild (<33%); 2: moderate (33–66%); 3: severe (>66%)) [23]. Based on previous descriptions, the scores for each parameter were summed, and the total tissue damage scores were calculated. Additionally, atretic follicles were defined according to the description of Osman et al. [30]. Thus, the follicles were defined according to the mean diameter: primordial (diameter < 20 µm), preantral (20–220 µm), small antral (221–310 µm) and large antral (311–370 µm). All of the ovarian sections were examined in a blind fashion by an experienced pathologist.

Immunohistochemical staining was conducted for the receptor area, with PCNA appearing as an indicator of follicular growth. In brief, following the de-paraffinization and dehydration of the sections, the sections were fixed at pH 6 in a microwave with 1 M citrate tamponade. After this procedure, hydrogen peroxide was applied for 15 min, and endogen peroxidase was stopped. After washing the lams at pH 7.4 four times for 4 min with PBS (phosphate-buffered saline), in order to forestall non-specific binding, Ultra V Block was utilized for 10 min. The sections were incubated with anti-PCNA Ab-1 for 60 min. After this process, sections were incubated with secondary antibodies following some procedures (biotinide anti-mouse/rabbit IgG, Diagnostic BioSystems, KP 50A, Pleasanton, CA, USA), Streptavidin Peroxidase Enzyme Complex Alkaline Phosphatase (20 min) (TS-060-AP, the Lab Vision Corporation, Fremont, CA, USA) and Fast Red Substrate System (TA-125-AF, the Lab Vision Corporation, USA). The sections were washed with PBS once more, and then Mayer’s hematoxylin was utilized, and lams were closed with Ultramount. The sections were evaluated under the Olympus Clinical Microscope BX45.

For biochemical analyses, the blood samples were obtained by cardiac puncture and transferred into EDTA-containing tubes. The plasma was separated by cold centrifugation (+4 °C) at 5000 rpm for 10 min. After centrifugation, the serum of blood samples was stored in a freezer at −80 °C for biochemical analyses. The TAS of supernatant fractions was evaluated using a novel automated measurement method developed by Erel. The results are expressed as μmol Trolox Eq/L [9,31]. The TOS of supernatant fractions was also evaluated using a novel automated measurement method developed by Erel. The assay is calibrated with hydrogen peroxide (H_2_O_2_) and the findings are expressed in terms of nmol H_2_O_2_ Eq/L [32,33]. OSI was calculated by the ratio of TOS to TAS. The formula of calculation for OSI: OSI (arbitrary unit) = TOS (μmol H_2_O_2_ Eq/L)/TAS (μmol Trolox Eq/L) [34].

### 2.4. Statistical Analysis

Statistical analysis was evaluated using GraphPad Software Inc., version 10.0 (GraphPad Inc., La Jolla, CA, USA). Means and standard deviations were used to describe numerical variables. To evaluate the distribution pattern of the data, the Kolmogorov–Smirnov test was used. The mean differences between groups were analyzed by Kruskal–Wallis and postdoc Dunn’s tests. The relationship between continuous variables was evaluated using Spearman’s correlation coefficient. *p* < 0.05 was considered significant for all statistical data.

## 3. Results

**Figure 1:**
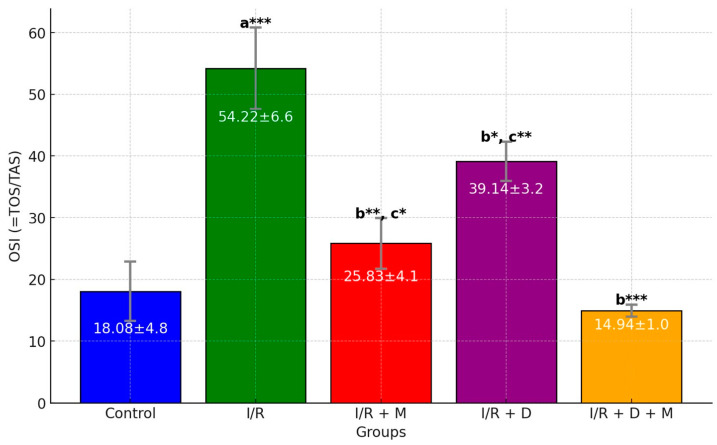
Bar graph of OSI (oxidative stress index) levels. ^a^, vs. control; ^b^, vs. I/R; ^c^: I/R + D + M. *: *p* < 0.05; **: *p* < 0.01; ***: *p* < 0.001. I/R: ischemia/reperfusion; M: mannitol; D: vitamin D.

The assessment of histopathological scores (PCNA) and oxidative stress parameters is presented in Table 1. In terms of PCNA, there was a substantial increase in group I/R + D + M as compared to the groups I/R, I/R + M and I/R + D (*p* < 0.05). Additionally, the cellular degeneration score, which shows acute tissue damage in the antral follicles, substantially increased in groups I/R as compared to other groups. There was a substantial increase in groups I/R + M, I/R + D and I/R + D + M as compared with the control group with regard to the average values of the TOS and OSI (*p* < 0.05). In the meantime, the average TOS and OSI values in group I/R + D + M were statistically lower than groups I/R, I/R + M and I/R + D (*p* < 0.001) (Table 1, Figure 1).

**Figure 2:**
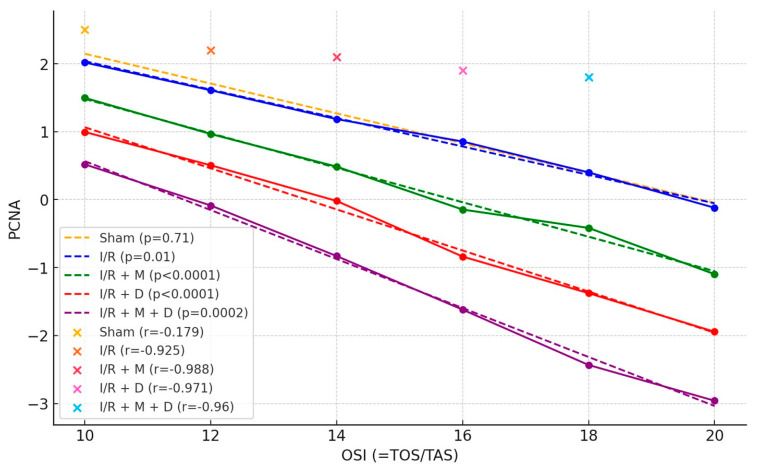
Correlation between OSI (oxidative stress index) and PCNA (proliferating cell nuclear antigen). I/R: ischemia/reperfusion; M: mannitol; D: vitamin D.

There was a strong negative correlation between OSI and proliferating cell nuclear antigen (PCNA) in groups I/R, I/R + M, I/R + D and I/R + M + D (r = −0.92, *p* = 0.01; r = −0.98, *p* < 0.0001; r = −0.98, *p* < 0.0001 and r = −0.96, *p* = 0.0002, respectively) (Figure 2).

**Figure 3:**
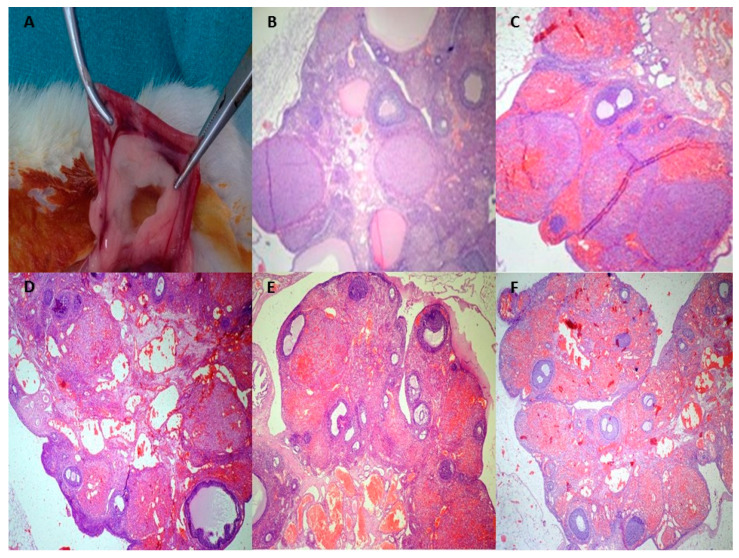
(**A**) Macroscopic view of adnexal tissue. (**B**) Sham group. Normal ovarian morphology (H&E ×100). (**C**) Detorsion group. A large amount of hemorrhage, congestion, inflammatory cell infiltration and decreased ovarian reserve (H&E ×100). (**D**) I/R + mannitol group. Moderate hemorrhage, inflammatory cell infiltration and cellular degeneration, as well as mild congestion, increased the primordial and primary number of follicles (H&E ×100). (**E**) I/R + vitamin D group. A small number of inflammatory cells infiltrated as well as mild hemorrhage, cellular degeneration, congestion and increased primary and preantral follicles (H&E ×100). (**F**) I/R + mannitol + vitamin D group. Mild hemorrhage, congestion, inflammatory cell infiltration, mild cellular degeneration and increased primary, primordial and preantral follicles (H&E ×100).

Additionally, the cellular degeneration score, which shows the acute tissue damage in the antral follicles, substantially increased in groups I/R, I/R + M, I/R + D and I/R + M + D as compared to the control group. Primordial and primary numbers of follicles in groups I/R + M, I/R + D, and I/R + M + D were found to be significantly higher in comparison with group I/R (*p* < 0.001). Likewise, preantral and antral follicle counts in groups I/R + M, I/R + D and I/R + M + D were substantially higher than those in group I/R (*p* < 0.01). Yet, primary, preantral and follicle counts increased in group I/R + D in comparison with group I/R + M + D (*p* < 0.01) (Table 2, Figure 3).

**Figure 4:**
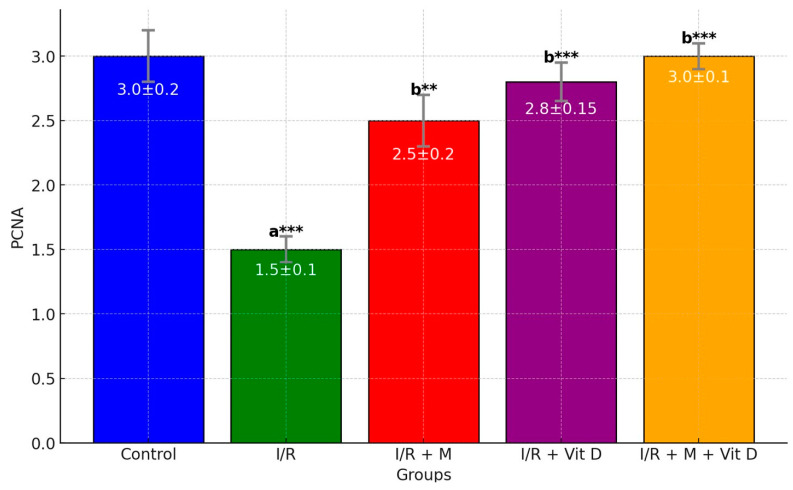
Bar graphs of PCNA levels. I/R: ischemia/reperfusion; M: mannitol; D: vitamin D. ^a^: vs. control; ^b^: vs. D; **: *p* < 0.01; ***: *p* < 0.001.

PCNA staining, which shows follicle viability, was observed to be lower in the detorsion group as compared to groups I/R + mannitol, I/R + D and I/R + mannitol + D (*p* < 0.001, *p* < 0.01 and *p* < 0.001, respectively) (Figure 4).

## 4. Discussion

To the authors’ knowledge, this is the first study to demonstrate the therapeutic and protective effects of combination treatment of vitamin D and mannitol in a rat ovarian torsion–detorsion model induced by I/R injury. In this study, it was observed that vitamin D and mannitol increased the level of PCNA in the rat ovarian I/R injury model, and they increased the number of reproductive follicles by decreasing the oxidative stress index.

Biochemical and morphological changes occur in ovarian tissues that were subjected to the ovarian torsion/detorsion model. Because of these changes, a number of changes were revealed in the ovarian reserve. To assess the ovarian reserve, usually the follicle count (primordial, primary, secondary and antral follicles) is examined [35]. In our study, we observed that the combination of mannitol and vitamin D provides a substantial increase in the number of primordial follicles; besides, it was observed that mannitol is more effective on the number of primary, secondary and antral follicles.

Follicle count in the evaluation of ovarian reserve may not be a reliable method alone. PCNA, showing the early stages of follicular development, gives more precise information on the assessment of ovarian reserve [36,37]. PCNA production, which is an early indicator of granulosa cell proliferation, increases in proportion to proliferation. The level of PCNA present in granulosa cells reduces with the passage of follicles to atresia [38,39,40]. In our study, it was detected that the PCNA level had higher significance in group I/R + mannitol + vitamin D according to the groups I/R, I/R + mannitol and I/R + vitamin D.

Inadequate blood flow and ischemia resulting from a lack of oxygen lead to decreased ATP production in tissues, increased lactic acid production and the accumulation of lipid peroxidase [27,41]. Depending on reperfusion, free oxygen radicals are produced, and active neutrophil accumulation occurs. Both of these activities allow for the release of reactive oxygen species [42,43]. These reactive oxygen species cause more cellular damage in mitochondrial and cell membranes due to lipid peroxidation in ischemic tissue [44,45]. Previous studies have shown that cellular damage is strongly correlated with the total score of oxidative stress [1,26]. In our study, it was found that instead of total scoring for cellular damage in ovarian tissue, ovarian reserve indicator PCNA and oxidative stress in between groups I/R, I/R + mannitol, I/R + vitamin D and I/R + mannitol + vitamin D were strongly correlated. In our study, both TAS and TOS values in I/R + mannitol + vitamin D were substantially lower in comparison with the groups I/R, I/R + mannitol and I/R + vitamin D. But there was no significant difference between the experimental groups in terms of TAS values.

Vitamin D is used to prevent and treat many organs in the presence of I/R injury, and the results of these treatments have been successful [11,15,16,25,46]. According to our knowledge, the impact of vitamin D on ovarian I/R injury has not been investigated by previous studies. Vitamin D protects the tissues with antioxidant, anti-inflammatory and apoptosis properties in ovarian I/R injury [47]. It has been known that mannitol actually causes postischemic edema with its own hyperosmolar feature [48]. However, Feige et al. found that, rather than the hyperosmolar feature of mannitol, the free radical scavenging feature was more effective in the myocardial I/R injury study [49]. In our study, it was found that the combination of mannitol with vitamin D before reperfusion led to a significant reduction in oxidative stress markers in ovarian I/R injury. At the same time, this combination provides a significant increase in the level of the ovarian reserve indicator PCNA.

The combination of vitamin D and mannitol likely enhances efficacy through several pharmacological mechanisms. Vitamin D activates the Nrf2/HO-1 antioxidant pathway, which reduces oxidative stress and inflammation by neutralizing reactive oxygen species (ROS) and downregulating pro-inflammatory cytokines. This pathway is critical for protecting tissues during I/R injury [17]. Its anti-inflammatory effects mitigate the inflammatory response typically seen in I/R injury by downregulating pro-inflammatory cytokines and upregulating anti-inflammatory cytokines [50]. Additionally, vitamin D promotes cell survival and inhibits apoptosis through various signaling pathways, such as the PI3K/Akt pathway, which is known to enhance cell viability and reduce cell death in ischemic conditions [51]. This protective effect is particularly important in mitigating the damage caused by I/R injury in ovarian tissues. Mannitol’s osmotic diuretic effect helps reduce tissue edema and improve microcirculation, which is essential for tissue recovery post-I/R injury. Furthermore, mannitol’s free radical scavenging properties help neutralize ROS, thus minimizing cellular and mitochondrial damage [52]. We have shown in our study that when these properties of mannitol are combined, they complement the effects of vitamin D, causing a more comprehensive reduction in oxidative stress and inflammation, thereby enhancing tissue recovery and the preservation of ovarian reserve.

Our study has some limitations. More biochemical and histopathological markers could have been included in the study.

## 5. Conclusions

In conclusion, it could be suggested that mannitol in combination with other vitamins is more effective than stand-alone use in an experimental rat I/R injury model. Also, it was observed that vitamin D and mannitol protect the ovarian reserve by reducing oxidative stress and increasing PCNA levels in ovarian I/R injury. However, more studies are needed to confirm the therapeutic use of these agents.

## Figures and Tables

**Table 1:** Comparison of oxidative stress and PCNA parameters in the groups (mean ± SD).

	Control	I/R	I/R + M	I/R + D	I/R + D + M
TAS (mmol/L)	1.32 ± 0.14	1.07 ± 0.01	1.09 ± 0.04	1.07 ± 0.05	1.07 ± 0.04
TOS (mmol/L)	24.90 ± 7.2	57.94 ± 6.6 ^a^**	29.02 ± 5.6 ^b,c^*	42.53 ± 4.5 ^c^**	16.16 ± 1.3 ^b^***
OSI (TOS/TAS)	18.08 ± 4.8	54.22 ± 6.6 ^a^***	25.83 ± 4.1 ^b^**^,c^*	39.14 ± 3.2 ^b^*^,c^**	14.94 ± 1.0 ^b^***
PCNA	2.91 ± 0.02	1.16 ± 0.02 ^a^***	2.40 ± 0.06 ^b^***^,c^*	2.02 ± 0.16 ^b,c^**	2.75 ± 0.05 ^b^***

TAS: total antioxidant status; TOS: total oxidant status; OSI: oxidative stress index; PCNA: proliferating cell nuclear antigens. I/R: ischemia/reperfusion; M: mannitol; D: vitamin D. ^a^: vs. control; ^b^: vs. I/R; ^c^: I/R + D + M. *: *p* < 0.05; **: *p* < 0.01; ***: *p* < 0.001.

**Table 2:** Comparison of follicle counts (fc) in groups (mean ± SD).

	Control	I/R	I/R + M	I/R + D	I/R + M + D
Primordial fc	5.28 ± 0.29	0.63 ± 0.04 ^a^***	3.40 ± 0.08 ^b^***	3.32 ± 0.11 ^b^***	3.71 ± 0.02 ^b^***
Primary fc	3.78 ± 0.07	0.68 ± 0.03 ^a^***	3.28 ± 0.06 ^b^***^, c^**	2.53 ± 0.04 ^b^***	2.42 ± 0.18 ^b^***
Preantral fc	6.10 ± 0.18	4.62 ± 0.20 ^a^***	6.35 ± 0.05 ^b^***^, c^**	6.92 ± 0.16 ^b,c^***	5.50 ± 0.13 ^b^**
Antral fc	5.51 ± 0.19	4.03 ± 0.09 ^a^***	7.15 ± 0.06 ^b,c^***	5.62 ± 0.04 ^b^***	5.88 ± 0.16 ^b^***

I/R: ischemia/reperfusion; M: mannitol; D: vitamin D. ^a^: vs. control; ^b^: vs. I/R; ^c^: I/R + M + D. **: *p* < 0.01; ***: *p* < 0.001.

## Data Availability

Data are available upon request to the corresponding author.

## Abbreviations

I/R	Ischemia–reperfusion injury
PCNA	Proliferating Cell Nuclear Antigen
TAS	Total antioxidant status
TOS	Total oxidant status
OSI	Oxidative stress index
